# Clinical and Growth Correlates of Retinopathy of Prematurity in Preterm infants with Surgical Necrotizing Enterocolitis and intestinal Perforation

**DOI:** 10.21203/rs.3.rs-3022247/v1

**Published:** 2023-06-06

**Authors:** Robin Riddick, Asha Meilstrup, Md Abu Yusuf Ansari, Jennifer Ware, David Zepponi, Andrea Smith, David Sawaya, Nils Mungan, Parvesh Mohan Garg

**Affiliations:** University of Mississippi Medical center; University of Mississippi Medical center; University of Mississippi Medial Center; University of Mississippi Medial Center; University of Mississippi Medial Center; University of Mississippi Medial Center; University of Mississippi Medial Center; University of Mississippi Medial Center; Wake Forest University

## Abstract

**Background:**

we sought to determine the clinical and growth parameters associated with retinopathy of prematurity (ROP) in infants with necrotizing enterocolitis (NEC) and spontaneous ileal perforation (SIP).

**Methods:**

Retrospective cohort study comparing clinical information before and following NEC/SIP onset in neonates with and without severe ROP (Type 1 and 2).

**Results:**

Those with severe ROP (32/109, 39.5%) had lower GA, BW, chorioamnionitis, later median onset of ROP diagnosis and received Penrose drain and had higher AKI, poor weight z scores, poor linear growth, longer duration of ventilation and higher FIo2 than those without ROP following NEC/SIP. The GA and diagnosis at later age remained significant for any ROP on multi regression modelling.

**Conclusion:**

The surgical NEC/SIP infants with severe ROP were more likely to be younger, smaller, had AKI, had higher oxygen exposure and poor weight gain and linear growth than those without severe ROP.

## Introduction

Necrotizing enterocolitis (NEC) is the most common acute gastrointestinal illness, affecting about 5–10% of preterm neonates with a birth weight ≤ 1500 grams [1, 2]. NEC remains a leading cause of morbidity due to NEC-associated severe systemic inflammatory response causing multi-organ dysfunction and mortality among preterm neonates and leads to increased health care burden.

Necrotizing enterocolitis is associated with necrosis, inflammation, hemorrhage and reparative changes on intestinal histopathological examination [3]. The hemorrhagic necrosis seen in infants with NEC is most likely due to anormal vasculature and neo angiogenesis in the intestine [4, 5]. The Retinopathy of prematurity is also associated with abnormal vascularization because of insulin like growth factor 1 (IGF-1) and vascular endothelial growth factor (VEGF) effect on the retinal angiogenesis [6]. In the Early Treatment for Retinopathy of Prematurity study, ROP developed in 68% of preterm infants < 1251 g and severe retinopathy of prematurity developed in almost 37% of the cases [7].A recent multicenter study has reported 12.8% of preterm infants born less than 28 weeks had severe ROP [8] and 2.5% of infants had bilateral blindness on the long-term follow-up. The surgical NEC and its timing of onset is a significant risk factor for the ROP development in preterm infants as shown in published reports [9, 10]. Surgical NEC is associated with dysbiosis and poor growth outcomes [11, 12]. Recent reports have reported the association between the altered gut microbiome and the retinopathy of prematurity [13]. Poor postnatal weight gain is reported to be a significant risk for severe ROP as well [14, 15].

There is no comprehensive study evaluating the surgical NEC/SIP characteristics and the growth data in detail in preterm infants with severe ROP before and following the NEC onset. In this single-center, retrospective cohort study, we sought to determine the clinical risk factors and growth characteristics that were associated with ROP before and after the surgical NEC onset in preterm infants.

## Methods

### Population and Study Design

**Figure 1:**
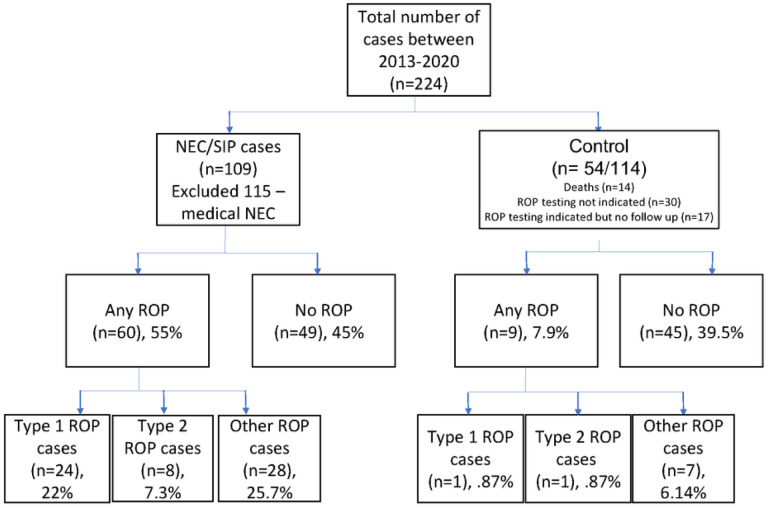
Legend not included with this version.

The study was conducted at the Level IV Neonatal unit at University of Mississippi Medical Center after IRB approval with approximately 1000 admissions/year including referrals from throughout the state. All neonates admitted between January 1, 2013 and June 2018, with a diagnosis of NEC (Bell stage III) were included in the study [16]. Neonates diagnosed with medical NEC were excluded from the analysis. The infants included in the study are summarized in Fig. 1.

### Demographic data

We collected demographic data including gestational age (GA), birth weight (BW), sex, appropriate for gestational age (AGA) status, race, out born status, mode of delivery, and Apgar scores ≤ 6 at 5 min. We also collected maternal variables including maternal pregnancy-induced hypertension (PIH), chorioamnionitis, and antenatal steroids.

Additional clinical information included mechanical ventilation exposure, presence of patent ductus arteriosus (PDA) and indomethacin/ibuprofen therapy for PDA treatment (before NEC), inotrope (dopamine) use 24 hours after NEC onset. In addition, we collected information on duration, Fio2 requirement, and mode of ventilation (invasive/noninvasive) before and following NEC. We also collected information on the blood culture-proven sepsis at the time of NEC onset, length of stay and mortality. The length of stay was defined as the total duration of hospitalization from the day of admission until discharge or death due to any cause before hospital discharge.

### NEC data

We recorded information on the age (in days) at the time of NEC diagnosis. The diagnosis of NEC was made based on characteristic radiographic findings including pneumatosis, portal venous gas, and pneumoperitoneum on abdominal X-ray. The frequency of medical and surgical NEC (Bell stage II and III) were also collected [16]. Neonates who died within 48 hours after NEC onset and massive bowel necrosis was found during laparotomy or autopsy were classified as having fulminant NEC. At our center, preterm infants with pneumoperitoneum who weigh less than 1 kg at NEC/SIP diagnosis and are hemodynamically unstable are treated first with a Penrose drain at the bedside but may later receive laparotomy. The timing of laparotomy after placement of Penrose drain was based on clinical deterioration.

### NEC *Histopathological Evaluation*

Hematoxylin and eosin-stained surgical resected intestinal tissue sections were evaluated for necrosis, inflammation, hemorrhage and reparative changes. A score of 0 was assigned when the exam appeared normal, 1 for 1–25% necrosis/ inflammation, 2 when 25–50% area involved, 3 when 50–75% area was affected, and 4 when > 75% changes were seen[17].

Post-operative information such as post-operative ileus days (defined as infants being NPO after bowel surgery), time to reach full feeds (≥ 120 ml/kg/day) and total parenteral nutrition days were also gathered. The surgical morbidity was classified as strictures, fistulas, wound dehiscence, surgical site infections (including abscesses), adhesions, and perforations.

#### Retinopathy of Prematurity Data:

We performed an analysis of 109 infants born during 2013–2018 that were admitted to the University of Mississippi Medical Center Neonatal Intensive Care Unit. ROP testing was indicated if the infant was born before 31 weeks gestational age or after 31 weeks if considered high risk. ROP was grouped into three categories: Type 1 ROP, type 2 ROP, and other ROP [10, 18]. Type 1 and type 2 ROP are the most severe types and usually require treatment. Any infant with plus disease was categorized as having type 1 ROP. Plus disease indicates dilated veins and tortuous arteries in the posterior pole of the eye. Type 2 ROP is any infant having stage 3 disease. All infants with type 1 ROP were treated with Laser photocoagulation or Avastin. Laser photocoagulation is an ablative treatment that targets avascular regions in order to decrease angiogenic factors such as Vascular Endothelial Growth factor (VEGF) and slow the growth of new abnormal blood vessels. Avastin (Bevacizumab) is a recombinant humanized monoclonal antibody that also targets VEGF and stops neovascularization.

### Kidney Function Data

We collected all serum creatinine measurements and daily urine output data starting the day before NEC diagnosis, at NEC onset, and up to 1 week after NEC diagnosis.

We defined AKI was defined using the modified neonatal AKI staging criteria as previously described in the kidney disease: Improving Global Outcomes (KDIGO) Clinical Practice Guideline [19–23].

### Bronchopulmonary dysplasia (BPD)data

BPD at 36 weeks corrected gestational age was classified as mild, moderate, and severe based on the oxygen requirement at assessment [24]. We collected data on the type of steroid (hydrocortisone/dexamethasone) used during the clinical course.

### Growth Outcome data

Time intervals include prior to developing NEC, during NEC treatment, post-NEC until anastomosis, after anastomosis, at 36 weeks chronological age, and at discharge. Anthropometric variables include weight, height, weight-for-length, head circumference, and respective z-scores. Sex-specific Fenton growth charts were used for infants less than 50 weeks old, and gender-specific WHO corrected for gestational age growth charts were used for infants greater than 50 weeks old.

### Brain injury data

MRI brain scans are routinely obtained at term equivalent age or before discharge home for all preterm infants weighing less than 1500 grams at birth. The MRI images were scored independently by two pediatric neuroradiologists We used a scoring system of eight scales for white and gray matter injury developed by Woodward et al. [25].The categories of white-matter abnormality were none (a score of 5 to 6), mild (a score of 7 to 9), moderate (a score of 10 to 12), and severe (a score of 13 to 15).

### Statistical Methods

In our study, we had analyzed the combined cohort of NEC/SIP and NEC alone. We analyzed all the continuous variables using the Mann-Whitney U-test and summarized with median and inter-quartile range (Quartile 1; Quartile 3). The categorical variables were tested using the Chi-squared test (or Fisher’s exact test when cell counts were below 5). The significant variables from the bivariate analyses were included in the multiple logistic regression. Adjusted odds ratios were reported as effect size along with 95% confidence interval and *P* value. Evaluations for significant multicollinearity led to birth weight and the corrected gestational age of ROP diagnosis being eliminated from the multivariable modeling process. *P* values less than 0.05 were considered as significant. Statistical analyses were performed in R Statistical Software (version 4.2.1; The R Foundation for Statistical Computing).

## Results

The demographic and clinical information of control (n = 54) and the infants with NEC/SIP (n = 109) is summarized in Table 1. Those with surgical NEC had significantly lower gestational age, lower birthweight, were less exposed to antenatal steroids (71% vs. 91.8%; p = 0.08), had onset of severe ROP at later day of life (60 days [44.0;75.0] vs. 45[35.0;48.]; p = < 0.001), had mainly Type 1 ROP (22% vs 1.8%).

### Combined Cohort NEC + SIP:

#### Any ROP

One hundred and nine infants (n = 109) with surgical necrotizing enterocolitis/SIP were included in the analysis. Sixty infants (60/109, 55%) were diagnosed with any ROP and 32/109 (29.3%) infants (22% Type 1 and 7.3% Type 2) had severe ROP.

Out of 60 cases, 24 (24/60, 40%) cases had type 1 ROP and 8 (8/60, 13.3%) cases had Type 2 ROP. 19 infants (19/60, 31.1%) were treated with Laser therapy and 12 infants (12/60,20%) received Avastin treatment. Six infants (6/60,10%) received both laser and Avastin treatment.

Infants with any ROP had significantly lower gestational age (24.4 weeks [23.5;25.4] vs. 27.3 [26.3;29.3], p = < 0.001) and lower median birth weight (665 grams [556;776] vs. 935 [700;1180], p = < 0.001) than those infants with sNEC/SIP without ROP. Those with ROP had lower frequency of portal venous gas (1/60,1.7% vs 6 /40, 12.2%; p = 0.045) on the abdominal Xray, received Penrose drain therapy more often (35/60, 59% vs 16/49,34%; p = 0.017) and had acute kidney injury by serum creatinine criteria more frequently (44 (78.6%) vs. 20 (46.5%); p = 0.002) than those infants with sNEC without ROP.

Those with any ROP had significantly higher exposure to pregnancy induced hypertension (11 (19.0%) vs. 20 (41.7%); p = 0.019) and chorioamnionitis (11/60 (19.0%) vs.1/49 (2.13%); p = 0.017) and Patent ductus arteriosus more often (75% vs. 55%; P = 0.048) and received indomethacin more frequently (22% vs.6.2%, p = 0.045) than those without any ROP. The data is shown in Table 2.

#### ROP Type 1 and 2 (NEC /SIP)

81 infants were included in the analysis. 32/81(39.5%) infants had type 1 and 2 ROP. Those with severe ROP had lower median gestational age (23.8 weeks [23.4;24.6] vs. 27.3 [26.3;29.], p = < 0.001), lower median birth weight (625 grams [512;710] vs.935 [700;1180]; p < 0.001) and had higher exposure to clinical chorioamnionitis (22.6% vs. 2.13%; p = < 0.006) than those without severe ROP. Those with severe ROP had later median onset of ROP diagnosis (63.0 days [47.0;77.2] vs. 29.0 [19.0;41.0]; p = < 0.001) and received Penrose drain therapy (19 (59.4%) vs.16 (34.0%); p = 0.046) more often and had higher acute kidney injury by creatinine more often (25 (86.2%) vs.20) than); p = 0.002) than those without ROP following NEC onset. Those with severe ROP had lower residual small bowel (70.0 cm [63.1;90.8] vs.90.8 [72.0;101]; p = 0.007), lower residual colon (22.7 cm [22.7;24.4] vs. 24.4 [22.7;36.0]; p = 0.003) than the other group, See Supplemental Table 1.

#### Oxygen and exposure ROP

Those with severe ROP were exposed to higher FiO2 at 7 days after birth (44.0% [30.0;57.0] vs. 25.0 [21.0;35.0]; p = 0.001) and were intubated longer (12.5 days [7.75;17.8] vs. 3.50 [1.00;4.75]; p < 0.001) before NEC and were exposed to a longer duration of invasive (47.0 days [33.0;70.0] vs. 16.0 days [8.50;45.8];p0.001), noninvasive ventilation (60.5 days [37.5;83.0]vs. 24.0 [9.00;42.5];p0.005) and higher FIo2 at 2 weeks (30% [25.0;38.0] vs. 25%[21.0;30.5];p = 0.007) following NEC compared to those without severe ROP. The data is shown in Supplemental Table 1.

There were no significant differences in the intestinal histopathology, postoperative features such as time to reach feeds and parenteral nutrition dependance, BPD, white matter and grey matter injury on brain MRI, length of stay and mortality in 2 groups. The data is shown in Table 3.

#### Growth outcomes and Severe ROP

**Figure 2:**
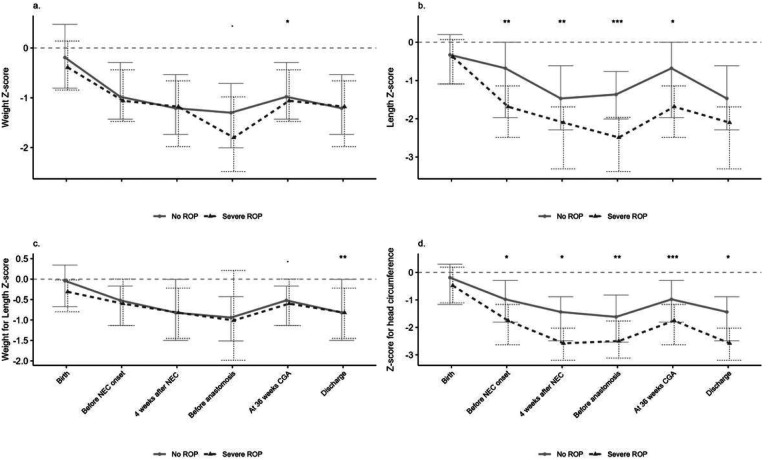
Legend not included with this version.

The preterm infants with severe (type 1 and type 2) ROP had significantly lower length and head circumference z scores before and following NEC. However, weight for length Z scores were significantly lower for infants with severe ROP compared to the other group. The data has been summarized in Fig. 2 and Supplemental Table 2.

#### Regression modelling

On multivariate regression modelling, gestational age (odds ratio 0.41 (95% CI 0.19–0.65); p = 0.004) and day of diagnosis of severe ROP (OR 1.09, 95% CI 1.05–1.17); p = 0.007) following NEC were most likely associated with ROP. The AKI, Fio2 requirement at 7 days of life and the duration of invasive ventilation following NEC were not significant. The data has been summarized in Table 5.

### NEC cohort:

77 infants were included in the analysis. 39/77 (50.6%) infants had any ROP. 15/39 (38.5%) infants had type 1 ROP and 6/39(15.4%) had type 2 ROP. 11/39 (28.2%) infants were treated with Laser therapy. 7/39 (17.9%) infants were treated with Avastin medication. 3 (7.69%) infants received both laser and Avastin treatment.

#### Any ROP

Preterm infants with any ROP had lower median gestational age (24.4 weeks [23.6;25.8] vs. 27.5 [26.4;29.6]; p < 0.001), lower median birth weight (670 grams [585;760] vs. 938 [688;]; p0]; p < 0.001) than those without any ROP. The data are shown in Supplemental Table 3.

#### Severe ROP (NEC cohort)

21/59 infants had severe ROP. Those with sever ROP had lower median gestational age (24 weeks [23.5;25.2] vs. 27.5 [26.4;29.6]; p < 0.001), lower median birth weight (640 grams [519;710] vs. 938 [688;1300], < 0.001), were diagnosed at later median of life (81days [69.0;94.0] vs. 43.5 [40.0;47.8];p < 0.001). The data are summarized in Supplemental Table 4.

#### Growth Outcomes

In the NEC cohort, the weight z scores and weight for length percentiles were significantly lower at 36 weeks corrected gestational age for the preterm infants with severe ROP. The length z scores were significantly lower before and 4 weeks following NEC onset and before the anastomosis in preterm. The data has been summarized in Fig. 2 and Supplemental Table 2.

## Discussion

In our cohort, 55% of infants with surgical NEC/SIP had any ROP and one third of infants had severe ROP. Type 1 ROP was more common than the Type 2 ROP. Almost one third of infants with severe ROP received laser treatment and one fifth of infants received avastin treatment. Only 10% of cases received both laser and the avastin treatment. Those with severe ROP were smaller, younger and were exposed to prenatal risk factors such as PIH, chorioamnionitis and postnatal risk factors including PDA, indomethacin, AKI, more Fio2, invasive and non- invasive mechanical ventilation more frequently. In addition, NEC infants with severe ROP had lower weight z scores and linear growth before and following the NEC onset.

The published reports have shown that the prematurity and the degree and duration of oxygen exposure influence the incidence of ROP in the preterm infants. In our NEC cohort, infants, infants with ROP were exposed to higher Fio2 and were ventilated invasively and non-invasively before and following the NEC onset in the univariate analysis. However the oxygen exposure was not a significant risk on the multivariate analysis, which may be explained due to: 1) other clinical factors play a key role than total oxygen exposure, 2) we failed to model the in -vivo oxygen saturation accurately 3) small sample size in the regression models as reported by Chen et al [26]. The studies have demonstrated the relationship between the oxygen and the ROP with phase I (hyperoxia-induced vasoconstriction and ischemic injury) and phase II (vascular endothelial growth factor–driven Vaso proliferation) of the disease [6].

In our cohort, the infants with severe ROP were younger (23.8 weeks vs. 27.3 weeks) and had lower birth weight (625 grams vs.935 grams) than those without severe ROP as reported in previous published report [27]. Studies done in discordant twin pairs have reported that gestational age is a better predictor of ROP severity than birth weight [28]. In a prospective study done in Australian and New Zealand Neonatal Network Darlow et al had reported that prematurity was the dominant risk factor, with infants with GA of <25 weeks having 20 times greater odds of severe ROP than infants with GA of 28 weeks. Birth weight for GA also had a “dose-response” effect; the more growth-restricted infants had greater risk, with infants below the 3rd percentile of weight for GA having 4 times greater odds of severe ROP than those between the 25th and 75th percentiles. Male gender was also a significant risk factor (odds ratio: 1.73; 95% confidence interval: 1.25–2.40) [29]. We did not see any sex difference in our study.

In our study , infants with severe ROP had poor linear growth at evidenced by lower length and head circumference z scores before and following NEC and were more exposed to chorioamnionitis which is similar to a recent prospective study reporting that slower length gain and maternal chorioamnionitis was associated with delayed regression and complete vascularization of retina in preterm infants [30].

In our NEC cohort, the weight z scores and weight for length percentiles were significantly lower at 36 weeks corrected gestational age for the preterm infants with severe ROP. As reported in published reports, the poor weight gain postnatally has been associated with severe ROP [31–33]. Postnatal weight gain is a surrogate indicator of insulin-like growth factor 1 (IGF-1), and a persistent lower serum IGF-1 in preterm infants is associated with poor weight gain [34]. Low serum IGF-1 also causes insufficient activation of retinal VEGF resulting in poor retinal vascular growth and the development of ROP [35].

In our cohort, AKI by creatinine following NEC onset was most likely associated with the severe ROP on bivariate analysis in infants with NEC/SIP which may most likely be explained due to systemic inflammation initiated secondary to NEC affecting kidneys and retina leading to multiple systemic morbidities. Several studies have shown that preterm infants with surgical NEC have severe white matter injury on the brain MRI, higher serum pro-inflammatory markers, and poor neurodevelopmental outcome at two years of corrected age [25, 36–39]. Animal studies have reported surgical NEC leads to systemic inflammation and causes neuronal injury via microglial activation, inflammatory pathway activation, and brain barrier disruption [40–43]. Mechanistically, we hypothesize that severe acute kidney injury in neonates with surgical NEC may exacerbate the injury by acting as a catalyst or modifier of retinal inflammation. Further studies are needed to understand the role of severe kidney injury with ROP in neonates with NEC.

Our study’s strengths include a comprehensive evaluation of clinical and growth factors most likely associated with severe ROP. Our study has important limitations. First, this was a single-center experience, reducing the study’s generalizability. Also in our cohort, most neonates with NEC were African American. While this is partly due to race distribution in Mississippi, this may also be related to adverse social determinants of health and genetic risk for NEC. Second, sample size limits our power to detect associations between clinical factors, NEC, and ROP. Finally, the small sample size coupled with multiple factors, outcomes, and comparisons may result in type I errors.

In conclusion, data shows that any ROP was diagnosed in 55% cases of infants with NEC/SIP and Type 1 ROP (38%) was more common and received both laser/ avastin therapy in 10% of cases, followed by type 2 in preterm infants with surgical NEC. Those with severe ROP were smaller, younger and were exposed to prenatal risk factors such as PIH, chorioamnionitis and postnatal risk factors including PDA, indomethacin, AKI, more Fio2, invasive and non- invasive mechanical ventilation more frequently. In addition, NEC infants with severe ROP had lower weight z scores and linear growth before and following the NEC onset. There is need to develop and implement strategies including aforementioned clinical risk factors to identify the NEC infants at greater risk of severe ROP to have better short term and the long-term eye outcomes. Weight gain, linear growth and nutrition should be closely monitored in preterm infants with surgical NEC/SIP at higher risk of severe ROP to improve the ophthalmic outcomes.

- supplementaltable1severeROPNECSIP.xlsx
- SupplementalTable2AGGROWTHCOMBINEDROP.docx
- SupplementalTable3NECCASESanyROP.xlsx
- COPYOFSUPPLEMENTAL4.1NECsevereROP.xlsx

## Figures and Tables

**Table 1:** Demographics and Clinical information of Control and Cases with NEC/SIP

	N	N = 163	Control N = 54	Surgical NEC/SIP = 109	p
Gestational Age (weeks, median, IQR)	163	26.4 [24.3;28.4]	27.4 [26.0;30.3]	25.4 [24.0;27.3]	**<0.001**
Birth Weight (g, median, IQR)	163	820 [655;1025]	990 [780;1303]	730 [620;940]	**<0.001**
Small for gestation, n (%)	163	10 (18.5%)	37 (34.9%)	47 (29.4%)	**0.049**
Male, n (%)	163	94 (57.7%)	27 (50.0%)	67 (61.5%)	0.22
Ethnicity, n (%)	160				**0.017**
African American		127 (79.4%)	43 (81.1%)	84 (78.5%)	
Caucasian		25 (15.6%)	5 (9.43%)	20 (18.7%)	
Hispanic		4 (2.50%)	4 (7.55%)	0 (0.00%)	
Other		4 (2.50%)	1 (1.89%)	3 (2.80%)	
Cesarean-section, n (%)	162	110 (67.9%)	35 (66.0%)	75 (68.8%)	0.861
Antenatal Steroid Use, n (%)	149	116 (77.9%)	45 (91.8%)	71 (71.0%)	**0.008**
Apgar score < 6 at 5 min, n (%)	160	38 (23.8%)	8 (15.1%)	30 (28.0%)	0.107
Day of life of severe ROP diagnosis (days), median (IQR)	162	49.0 [42.0;68.8]	45.0 [35.0;48.0]	60.0 [44.0;75.0]	**<0.001**
Corrected gestational age of severe ROP diagnosis (days), median (IQR)	163	34.3 [33.0;36.0]	34.0 [32.9;35.1]	34.4 [33.1;36.1]	0.064
Type 1 ROP n (%)	163	25 (15.3%)	1 (1.85%)	24 (22.0%)	**0.002**
Type 2 ROP n (%)	163	9 (5.52%)	1 (1.85%)	8 (7.34%)	0.274
No ROP n (%)	163	94 (57.7%)	45 (83.3%)	49 (45.0%)	**<0.001**
Laser, n (%)	163	20 (12.3%)	1 (1.85%)	19 (17.4%)	**0.009**
Avastin, n (%)	163	12 (7.36%)	0 (0.00%)	12 (11.0%)	**0.009**
Both, n (%)	163	6 (3.68%)	0 (0.00%)	6 (5.50%)	0.179
Length of Stay (days, median ± SD)	162	97.0 [65.5;165]	77.0 [63.0;99.0]	117 [72.0;171]	**0.001**
Death, n (%)	163	39 (23.9%)	2 (3.70%)	37 (33.9%)	**<0.001**

**Table 2:** Demographic and clinical information in infants with any ROP and no ROP in NEC/SIP cohort

	*N = 109*	*No ROP, N = 49*	*ROP, N = 60*	*p*
Day of life at no ROP (days), median (IQR)	47.0 [42.0;57.0]	44.0 [40.0;56.0]	52.0 [43.0;57.2]	**0.017**
Day of life of severe ROP diagnosis (days), median (IQR)	60.0 [44.0;75.0]	44.0 [40.0;56.0]	70.5 [60.8;87.0]	**<0.001**
Corrected gestational age of severe ROP diagnosis (days), median (IQR)	34.4 [33.1;36.1]	33.7 [32.6;35.2]	34.8 [33.7;37.0]	**0.015**
Type 1 ROP n (%)	24 (22.0)	0 (0.00)	24 (40.0)	**<0.001**
Type 2 ROP n (%)	8 (7.34)	0 (0.00)	8 (13.3)	**0.008**
No ROP n (%)	49 (45.0)	49 (100)	0 (0.00)	**<0.001**
Laser, n (%)	19 (17.4)	0 (0.00)	19 (31.7)	**<0.001**
Avastin, n (%)	12 (11.0)	0 (0.00)	12 (20.0)	**0.003**
Both, n (%)	6 (5.50)	0 (0.00)	6 (10.0)	**0.032**
**Prenatal information**					
Pregnancy-induced hypertension, n (%)	31 (29.2)	20 (41.7)	11 (19.0)	**0.019**
Chronic hypertension, n (%)	15 (15.8)	7 (15.9)	8 (15.7)	0.99
Chorioamnionitis, n (%)	12 (11.4)	1 (2.13)	11 (19.0)	**0.017**
Antenatal steroids, n (%)	71 (71.0)	30 (66.7)	41 (74.5)	0.521
**Infant demographics**					
Gestational age (weeks) (median [IQR])	25.4 [24.0;27.3]	27.3 [26.3;29.3]	24.4 [23.5;25.4]	**<0.001**
Birth weight (g) (median [IQR])	730 [620;940]	935 [700;1180]	665 [556;776]	**<0.001**
Small for gestational age, n (%)					
Male, n (%)	67 (61.5)	34 (69.4)	33 (55.0)	0.181
Race, n (%)				0.319
African American	84 (78.5)	40 (83.3)	44 (74.6)	
Caucasian	20 (18.7)	8 (16.7)	12 (20.3)	
Other	3 (2.80)	0 (0.00)	3 (5.08)	
Vaginal delivery, n (%)	34 (31.2)	16 (32.7)	18 (30.0)	0.929
Apgar score < 6 at 5 min, n (%)	30 (28.0)	4 (8.33)	26 (44.1)	**<0.001**
Out born, n (%)	69 (63.3)	28 (57.1)	41 (68.3)	0.314
**Infant medical information prior to NEC**					
Patent ductus arteriosus, n (%)	72 (66.1)	27 (55.1)	45 (75.0)	**0.048**
Patent ductus arteriosus, indomethacin, n (%)	16 (15.0)	3 (6.25)	13 (22.0)	**0.045**
Platelet transfusion before NEC, n (%)	78 (76.5)	36 (75.0)	42 (77.8)	0.923
Red blood cell transfusion before NEC, n (%)	85 (94.4)	39 (92.9)	46 (95.8)	0.661
**Postoperative systemic course**					
24 h Ionotropic support, n (%)	76 (73.1)	30 (63.8)	46 (80.7)	0.088
AKI by serum creatinine, n (%)				**0.006**
Normal	35 (35.4)	23 (53.5)	12 (21.4)	
Stage 1	23 (23.2)	5 (11.6)	18 (32.1)	
Stage 2	20 (20.2)	8 (18.6)	12 (21.4)	
Stage 3	21 (21.2)	7 (16.3)	14 (25.0)	
AKI by urine output, n (%)				0.986
Normal	54 (55.1)	23 (57.5)	31 (53.4)	
Stage 1	6 (6.12)	2 (5.00)	4 (6.90)	
Stage 2	27 (27.6)	11 (27.5)	16 (27.6)	
Stage 3	11 (11.2)	4 (10.0)	7 (12.1)	
Central line present (days) (median [IQR])	60.0 [38.0;99.0]	60.0 [43.0;87.0]	53.5 [36.2;108]	0.991
Positive blood culture sepsis, n (%)	36 (33.0)	16 (32.7)	20 (33.3)	0.99
CRP on day of NEC onset (median [IQR])	8.70 [3.20;17.7]	12.6 [4.40;19.0]	8.00 [2.98;17.7]	0.575
CRP at 1 week after NEC onset (median [IQR])	4.60 [2.50;7.70]	5.80 [3.00;13.4]	4.45 [2.45;6.62]	0.173
**Hematology data**				
Any packed red cell Transfusion before NEC	85 (94.4)	39 (92.9)	46 (95.8)	0.661
Hematocrit before NEC onset	34.0 [30.2;38.2]	34.4 [29.9;39.0]	33.8 [30.6;37.9]	0.655
Packed red cell Transfusion 48 before NEC	18 (25.7)	5 (18.5)	13 (30.2)	0.418
Packed red cell Transfusion 48h after NEC	77 (78.6)	37 (78.7)	40 (78.4)	1.000
Platelet transfusion before NEC	78 (76.5)	36 (75.0)	42 (77.8)	0.923
Platelet transfusion 48h after NEC	41 (45.1)	20 (44.4)	21 (45.7)	1.000
Cholestasis at NEC onset, n (%)	61 (69.3)	25 (69.4)	36 (69.2)	0.99
Length of stay (days) (median [IQR])	117 [72.0;171]	138 [47.0;171]	116 [75.8;172]	0.951
Death, n (%)	37 (33.9)	21 (42.9)	16 (26.7)	0.116

**Table 3:** NEC features, Oxygen and Ventilation data

	N = 109	No ROP, N = 49	Any ROP, N = 60	p Value
Clinical Presentation, n (%)				**0.004**
Abdominal Distension	98 (91.6)	41 (83.7)	57 (98.3)	
Bloody Stools	6 (5.61)	6 (12.2)	0 (0.00)	
Feeding Intolerance	3 (2.80)	2 (4.08)	1 (1.72)	
Pneumatosis	42 (38.9)	22 (44.9)	20 (33.9)	0.332
Pneumoperitoneum	62 (57.4)	25 (51.0)	37 (62.7)	0.304
Portal Venous Gas	7 (6.48)	6 (12.2)	1 (1.69)	**0.045**
Age of NEC Onset (days), median (IQR)	11.0 [7.00;23.0]	12.0 [5.00;23.0]	11.0 [7.75;25.0]	0.463
Fulminant NEC, n (%)	10 (9.26)	4 (8.16)	6 (10.2)	0.99
Present of Penrose Drain, n (%)	51 (48.1)	16 (34.0)	35 (59.3)	**0.017**
Length of Bowel Resected (cm), median (IQR)	10.7 [4.27;27.4]	15.0 [5.35;36.6]	9.70 [3.50;21.5]	0.052
Region of Bowel Resected, n (%)				0.742
Small Bowel Resected	69 (69.0)	33 (71.7)	36 (66.7)	
Ileostomy, n (%)	62 (56.9)	23 (46.9)	39 (65.0)	0.089
Colostomy, n (%)	11 (10.1)	2 (4.08)	9 (15.0)	0.107
Jejunostomy, n (%)	34 (31.2)	19 (38.8)	15 (25.0)	0.181
Combined large and Small Bowel Resected	31 (31.0)	13 (28.3)	18 (33.3)	
Presence of Ileocecal Valve, n (%)	76 (72.4)	32 (66.7)	44 (77.2)	0.326
Postoperative ileus days (days) (median [IQR])	13.0 [11.0;17.5]	12.5 [11.0;17.2]	13.0 [10.5;17.0]	0.685
Postoperative day at starting enteral feedings (days) (median [IQR])	14.0 [12.0;18.0]	14.0 [12.0;18.0]	14.0 [11.8;18.8]	0.777
Day attainment of full enteral feedings (120 ml/kg) (median [IQR])	65.5 [32.0;92.2]	62.0 [41.0;93.0]	69.0 [29.0;89.0]	0.802
Duration of parenteral nutrition (days) (median [IQR])	81.0 [38.0;118]	76.5 [36.2;120]	86.0 [39.0;118]	0.600
**Surgical Morbidity (Infection, Adhesions, Strictures, Dehiscence), n (%)**	78 (71.6)	36 (73.5)	42 (70.0)	0.852
More than One Surgical Morbidity (Infection, Adhesions, Strictures, Dehiscence), n (%)				
Adhesions, n (%)	56 (51.4)	22 (44.9)	34 (56.7)	0.303
Wound Dehiscence, n (%)	28 (25.7)	11 (22.4)	17 (28.3)	0.632
Wound Infection, n (%)	14 (12.8)	10 (20.4)	4 (6.67)	0.065
Stricture, n (%)	12 (11.0)	5 (10.2)	7 (11.7)	0.99
Fistula, n (%)	13 (11.9)	5 (10.2)	8 (13.3)	0.838
Compartment Syndrome, n (%)	8 (7.34)	2 (4.08)	6 (10.0)	0.291
Intestinal Failure, n (%)	32 (29.4)	11 (22.4)	21 (35.0)	0.222
**Oxygen and ventilation data**				
Day 7 Fio2	30.0 [21.5;39.0]	21.0 [21.0;30.0]	35.0 [29.0;46.5]	**0.001**
Fio2 Admission Out born	44.5 [29.0;68.8]	32.0 [27.5;43.8]	51.0 [30.0;75.8]	0.092
Invasive ventilation duration before NEC	7.00 [4.00;13.8]	3.50 [1.00;4.75]	8.50 [6.50;15.0]	**<0.001**
Non-invasive duration before NEC	8.00 [3.25;15.5]	9.50 [6.25;16.2]	3.50 [2.75;10.0]	0.191
Fio2 7 days before NEC	25.5 [21.0;39.5]	23.0 [21.0;33.2]	28.0 [22.8;39.5]	0.281
Invasive vent duration after NEC (days)	39.0 [12.0;57.0]	16.0 [8.50;45.8]	45.0 [18.0;65.0]	**0.001**
Non-invasive duration after NEC	46.0 [22.0;73.0]	24.0 [9.00;42.5]	62.0 [38.5;99.5]	**0.001**
Fio2 after 2 weeks NEC	29.0 [23.0;36.0]	25.0 [21.0;30.5]	30.0 [25.0;38.0]	**0.002**
Ileostomy, n (%)	62 (56.9)	23 (46.9)	39 (65.0)	0.089
Colostomy, n (%)	11 (10.1)	2 (4.08)	9 (15.0)	0.107
Jejunostomy, n (%)	34 (31.2)	19 (38.8)	15 (25.0)	0.181
BPD, n (%)				0.051
No BPD	12 (15.0)	8 (23.5)	4 (8.70)	
Mild	9 (11.2)	1 (2.94)	8 (17.4)	
Moderate	19 (23.8)	10 (29.4)	9 (19.6)	
Severe	40 (50.0)	15 (44.1)	25 (54.3)	
Postnatal use of steroids, n (%)	68 (63.0)	29 (59.2)	39 (66.1)	0.588

**Table 4:** Multinomial regression modelling for any ROP in infants with NEC/SIP

Predictors	aOR	95% CI	P value
Fio2 at day 7 of life	1.04	0.99–1.09	0.138
Gestational age	0.41	0.19–0.65	**0.004**
Day of severe ROP diagnosis after birth	1.09	1.03–1.17	**0.007**
Invasive ventilation after birth	0.99	0.96–1.02	0.429
AKI by serum creatinine	5.24	0.76–47.61	0.105

## References

[1] Neu J. and Walker W.A., Necrotizing enterocolitis. N Engl J Med, 2011. 364(3): p. 255–64. 21247316 10.1056/NEJMra1005408 PMC3628622

[2] Sankaran K., , Variations in incidence of necrotizing enterocolitis in Canadian neonatal intensive care units. J Pediatr Gastroenterol Nutr, 2004. 39(4): p. 366–72. 15448426 10.1097/00005176-200410000-00012

[3] Garg P.M., , Incomplete resection of necrotic bowel may increase mortality in infants with necrotizing enterocolitis. Pediatr Res, 2021. 89(1): p. 163–170. 32438367 10.1038/s41390-020-0975-6 PMC7679278

[4] Garg P.M., , Clinical determinants and impact of hemorrhagic lesions on intestinal pathology in preterm infants with surgical necrotizing enterocolitis. J Neonatal Perinatal Med, 2023. 16(1): p. 119–128. 36565070 10.3233/NPM-221116 PMC10324376

[5] Garg P.M., , Clinical and histopathological correlates of intestinal repair in preterm infants following surgical necrotizing enterocolitis. J Matern Fetal Neonatal Med, 2022. 35(26): p. 10565–10576. 36261134 10.1080/14767058.2022.2134773 PMC10363770

[6] Hartnett M.E. and Penn J.S., Mechanisms and management of retinopathy of prematurity. N Engl J Med, 2012. 367(26): p. 2515–26. 23268666 10.1056/NEJMra1208129 PMC3695731

[7] Good W.V., , The incidence and course of retinopathy of prematurity: findings from the early treatment for retinopathy of prematurity study. Pediatrics, 2005. 116(1): p. 15–23. 15995025 10.1542/peds.2004-1413

[8] Bell E.F., , Mortality, In-Hospital Morbidity, Care Practices, and 2-Year Outcomes for Extremely Preterm Infants in the US, 2013–2018. Jama, 2022. 327(3): p. 248–263. 35040888 10.1001/jama.2021.23580 PMC8767441

[9] Yucel O.E., , Incidence and risk factors for retinopathy of prematurity in premature, extremely low birth weight and extremely low gestational age infants. BMC Ophthalmol, 2022. 22(1): p. 367. 36096834 10.1186/s12886-022-02591-9 PMC9469514

[10] Fundora J.B., , Association of Surgical Necrotizing Enterocolitis and its Timing with Retinopathy of Prematurity. Am J Perinatol, 2021. 10.1055/s-0041-1733785 PMC8939240 34344041

[11] Pammi M., , Intestinal dysbiosis in preterm infants preceding necrotizing enterocolitis: a systematic review and meta-analysis. Microbiome, 2017. 5(1): p. 31. 28274256 10.1186/s40168-017-0248-8 PMC5343300

[12] Bell M., , Neurodevelopmental and Growth Outcomes of Extremely Preterm Infants with Short Bowel Syndrome. J Pediatr, 2021. 230: p. 76–83.e5. 33246015 10.1016/j.jpeds.2020.11.026 PMC8861973

[13] Zhang J.Y., Greenwald M.J., and Rodriguez S.H., Gut Microbiome and Retinopathy of Prematurity. Am J Pathol, 2023. 10.1016/j.ajpath.2023.01.013 36780985

[14] Wongnophirun A., , Association between severe retinopathy of prematurity and postnatal weight gain in very low-birthweight infants at Chiang Mai University Hospital, Thailand. Paediatr Int Child Health, 2020. 40(2): p. 85–91. 31272307 10.1080/20469047.2019.1631588

[15] Aydemir O., , Adjusted poor weight gain for birth weight and gestational age as a predictor of severe ROP in VLBW infants. Eye (Lond), 2011. 25(6): p. 725–9. 21378993 10.1038/eye.2011.29 PMC3178121

[16] Bell M.J., , Neonatal necrotizing enterocolitis. Therapeutic decisions based upon clinical staging. Ann Surg, 1978. 187(1): p. 1–7. 413500 10.1097/00000658-197801000-00001 PMC1396409

[17] Remon J.I., , Depth of bacterial invasion in resected intestinal tissue predicts mortality in surgical necrotizing enterocolitis. J Perinatol, 2015. 35(9): p. 755–62. 25950918 10.1038/jp.2015.51 PMC4552605

[18] Revised indications for the treatment of retinopathy of prematurity: results of the early treatment for retinopathy of prematurity randomized trial. Arch Ophthalmol, 2003. 121(12): p. 1684–94. 14662586 10.1001/archopht.121.12.1684

[19] Selewski D.T., , Neonatal Acute Kidney Injury. Pediatrics, 2015. 136(2): p. e463–73. 26169430 10.1542/peds.2014-3819

[20] Jetton J.G., , Incidence and outcomes of neonatal acute kidney injury (AWAKEN): a multicentre, multinational, observational cohort study. Lancet Child Adolesc Health, 2017. 1(3): p. 184–194. 29732396 10.1016/S2352-4642(17)30069-X PMC5933049

[21] Jetton J.G., , Assessment of Worldwide Acute Kidney Injury Epidemiology in Neonates: Design of a Retrospective Cohort Study. Front Pediatr, 2016. 4: p. 68. 27486571 10.3389/fped.2016.00068 PMC4950470

[22] Jetton J.G. and Askenazi D.J., Acute kidney injury in the neonate. Clin Perinatol, 2014. 41(3): p. 487–502. 25155722 10.1016/j.clp.2014.05.001

[23] Zappitelli M., , Developing a neonatal acute kidney injury research definition: a report from the NIDDK neonatal AKI workshop. Pediatr Res, 2017. 82(4): p. 569–573. 28604760 10.1038/pr.2017.136 PMC9673450

[24] Jobe A.H. and Bancalari E., Bronchopulmonary dysplasia. Am J Respir Crit Care Med, 2001. 163(7): p. 1723–9. 11401896 10.1164/ajrccm.163.7.2011060

[25] Woodward L.J., , Neonatal MRI to predict neurodevelopmental outcomes in preterm infants. N Engl J Med, 2006. 355(7): p. 685–94. 16914704 10.1056/NEJMoa053792

[26] Chen J.S., , Quantification of Early Neonatal Oxygen Exposure as a Risk Factor for Retinopathy of Prematurity Requiring Treatment. Ophthalmol Sci, 2021. 1(4): p. 100070. 36275192 10.1016/j.xops.2021.100070 PMC9562374

[27] Sabri K., , Retinopathy of Prematurity: A Global Perspective and Recent Developments. Pediatrics, 2022. 150(3). 10.1542/peds.2021-053924 35948728

[28] Wang Z.H., Li Y.Y., and Liu Z.M., Birth weight and gestational age on retinopathy of prematurity in discordant twins in China. Int J Ophthalmol, 2014. 7(4): p. 663–7. 25161939 10.3980/j.issn.2222-3959.2014.04.14 PMC4137203

[29] Darlow B.A., , Prenatal risk factors for severe retinopathy of prematurity among very preterm infants of the Australian and New Zealand Neonatal Network. Pediatrics, 2005. 115(4): p. 990–6. 15805375 10.1542/peds.2004-1309

[30] Schoephoerster J., , Identification of clinical factors associated with timing and duration of spontaneous regression of retinopathy of prematurity not requiring treatment. J Perinatol, 2023. 10.1038/s41372-023-01649-w 36973383

[31] Sethi N.K., , Study to evaluate the relation between weight gain in infants and occurrence of retinopathy of prematurity. Indian J Ophthalmol, 2023. 71(3): p. 890–894. 36872704 10.4103/ijo.IJO_1538_22 PMC10229907

[32] Wallace D.K., , Poor postnatal weight gain: a risk factor for severe retinopathy of prematurity. J aapos, 2000. 4(6): p. 343–7. 11124668 10.1067/mpa.2000.110342

[33] Lin L. and Binenbaum G., Postnatal weight gain and retinopathy of prematurity. Semin Perinatol, 2019. 43(6): p. 352–359. 31221520 10.1053/j.semperi.2019.05.008

[34] Löfqvist C., , Longitudinal postnatal weight and insulin-like growth factor I measurements in the prediction of retinopathy of prematurity. Arch Ophthalmol, 2006. 124(12): p. 1711–8. 17159030 10.1001/archopht.124.12.1711

[35] Smith L.E., , Regulation of vascular endothelial growth factor-dependent retinal neovascularization by insulin-like growth factor-1 receptor. Nat Med, 1999. 5(12): p. 1390–5. 10581081 10.1038/70963

[36] Hintz S.R., , Neuroimaging and neurodevelopmental outcome in extremely preterm infants. Pediatrics, 2015. 135(1): p. e32–42. 25554820 10.1542/peds.2014-0898 PMC4279063

[37] Shin S.H., , Surgical Necrotizing Enterocolitis versus Spontaneous Intestinal Perforation in White Matter Injury on Brain Magnetic Resonance Imaging. Neonatology, 2016. 110(2): p. 148–54. 27105356 10.1159/000444387

[38] Merhar S.L., , Brain magnetic resonance imaging in infants with surgical necrotizing enterocolitis or spontaneous intestinal perforation versus medical necrotizing enterocolitis. J Pediatr, 2014. 164(2): p. 410–2.e1. 24210927 10.1016/j.jpeds.2013.09.055

[39] Maheshwari A., , Cytokines associated with necrotizing enterocolitis in extremely-low-birth-weight infants. Pediatr Res, 2014. 76(1): p. 100–8. 24732104 10.1038/pr.2014.48 PMC4062583

[40] Adén U., , Systemic inflammation sensitizes the neonatal brain to excitotoxicity through a pro-/anti-inflammatory imbalance: key role of TNFalpha pathway and protection by etanercept. Brain Behav Immun, 2010. 24(5): p. 747–58. 19861157 10.1016/j.bbi.2009.10.010

[41] Brunse A., Abbaspour A., and Sangild P.T., Brain Barrier Disruption and Region-Specific Neuronal Degeneration during Necrotizing Enterocolitis in Preterm Pigs. Dev Neurosci, 2018. 40(3): p. 198–208. 29874640 10.1159/000488979

[42] Niño D.F., , Cognitive impairments induced by necrotizing enterocolitis can be prevented by inhibiting microglial activation in mouse brain. Sci Transl Med, 2018. 10(471). 10.1126/scitranslmed.aan0237 PMC8170511 30541786

[43] Biouss G., , Experimental necrotizing enterocolitis induces neuroinflammation in the neonatal brain. J Neuroinflammation, 2019. 16(1): p. 97. 31077225 10.1186/s12974-019-1481-9 PMC6511222

